# A circulating microRNA panel enhances the diagnosis of cholangiocarcinoma

**DOI:** 10.1371/journal.pone.0333279

**Published:** 2025-09-25

**Authors:** Aye Myat Mon, Kitti Intuyod, Sirinapha Klungsaeng, Apinya Jusakul, Arunnee Sangka, Vor Luvira, Chawalit Pairojkul, Tullayakorn Plengsuriyakarn, Kesara Na-Bangchang, Somchai Pinlaor, Porntip Pinlaor

**Affiliations:** 1 Medical Technology Program, Faculty of Associated Medical Sciences, Khon Kaen University, Khon Kaen, Thailand; 2 Cholangiocarcinoma Research Institute, Faculty of Medicine, Khon Kaen University, Thailand; 3 Department of Pathology, Faculty of Medicine, Khon Kaen University, Khon Kaen, Thailand; 4 Department of Parasitology, Faculty of Medicine, Khon Kaen University, Khon Kaen, Thailand; 5 Centre for Research and Development of Medical Diagnostic Laboratories, Faculty of Associated Medical Sciences, Khon Kaen University, Khon Kaen, Thailand; 6 Department of Surgery, Faculty of Medicine, Khon Kaen UniversityKhon Kaen, Thailand; 7 Graduate Program in Bioclinical Sciences, Chulabhorn International College of Medicine, Thammasat University (Rangsit Campus), Center of Excellence in Pharmacology and Molecular Biology of Malaria and Cholangiocarcinoma, Thammasat University (Rangsit Campus), Pathumthani, Thailand; Institute of Cytology and Genetics SB RAS: FIC Institut citologii i genetiki Sibirskogo otdelenia Rossijskoj akademii nauk, RUSSIAN FEDERATION

## Abstract

**Background and aim:**

Cholangiocarcinoma (CCA), a malignancy with a high incidence in regions endemic for the liver fluke *Opisthorchis viverrini*, is frequently characterized by alterations in the p53 tumor-suppressor gene, a process often modulated by microRNAs. Our study aimed to identify circulating miRNAs as potential diagnostic biomarkers for CCA.

**Methods:**

We sequenced small RNAs to identify differentially expressed microRNAs (miRNAs) in two CCA cell lines (one p53-mutant, one p53-wildtype) relative to an immortalized cholangiocyte cell line. Candidate miRNAs exhibiting significant upregulation with a log2 fold change > 4.5 were selected for validation via RT-qPCR in serum samples from patients with CCA. The diagnostic utility of these serum miRNAs was subsequently evaluated using receiver operating characteristic (ROC) analysis to assess their ability to distinguish CCA from normal controls and hepatocellular carcinoma (HCC). This performance was assessed for the miRNAs as standalone markers and in combination with conventional tumor markers.

**Results:**

Analysis of miRNA expression profiles identified seven upregulated candidates for validation. Among these, serum levels of miR-99a-5p, miR-516a-5p, and miR-526b-5p were significantly elevated in patients with CCA compared to both normal controls (all area under the curve, AUC > 0.79, p < 0.0001) and patients with HCC (all AUC > 0.80, p < 0.0001). A diagnostic panel combining these three miRNAs demonstrated high accuracy in distinguishing CCA from normal controls (AUC = 0.899) and from HCC (AUC = 0.937). The panel’s performance was further enhanced by incorporating conventional tumor markers, achieving an AUC of 0.928 when combined with CA19−9, and rising to 0.959 with the inclusion of both CA19−9 and CEA.

**Conclusions:**

These findings indicate that a panel comprising miR-99a-5p, miR-516a-5p, and miR-526b-5p, alone or with established tumor markers, offers high accuracy as a minimally invasive diagnostic tool for CCA, and can effectively distinguish it from HCC.

## Introduction

Cholangiocarcinoma (CCA) is a cancer of bile-duct epithelial cells. Incidence and mortality rates of CCA have been increasing worldwide over the past decades [1]. The regional incidence of CCA varies, reaching its peak in northeastern Thailand, where opisthorchiasis, caused by the carcinogenic liver fluke *Opisthorchis viverrini* (Ov), is most prevalent [2]. The diagnosis of CCA relies on laboratory tests and imaging techniques, such as CT, MRI, echo-endoscopy, or liver biopsy. The blood tumor markers commonly used, such as carbohydrate antigen 19−9 (CA19−9) and carcinogenic embryonic antigen (CEA) [3], have certain limitations in terms of sensitivity and specificity when it comes to identifying and monitoring CCA [4]. The absence of precise diagnostic methods is one of the reasons for delayed diagnosis. These factors, along with the ineffectiveness of current chemotherapeutic drugs, lead to the relatively low 5-year survival rate of CCA patients [5]. Thus, there is an urgent need to identify efficient biomarkers for the diagnosis of CCA.

The nature of CCA shows a high degree of heterogeneity [6]. Opisthorchiasis-associated CCA is characterized by specific mutational changes of cancer-related genes, including the p53 tumor suppressor gene, suppressor of mothers against decapentaplegic family member 4, and AT-rich interaction domain 1A [7–9]. Notably, alterations in p53 not only disrupt its tumor-suppressive functions [10] but also affect the expression and maturation of microRNAs (miRNAs) which play critical roles in cellular regulation and tumorigenesis [11]. MiRNAs are short, non-coding RNAs that modulate protein expression through the suppression of messenger RNA translation, influencing various physiological and pathological processes, including cell proliferation, differentiation, and apoptosis [12].

Circulating miRNAs found in biofluids such as plasma, serum, and urine are increasingly of interest as potential biomarkers for various diseases, including cancer [13]. When analyzed in serum samples, specific miRNA panels could distinguish between cancerous and non-cancerous conditions and differentiate among various tumor types with high accuracy. Studies have proposed miRNA panels as promising diagnostic biomarkers for several cancers, including bladder [14], pancreatic [15], colorectal [16], hepatocellular [17], laryngeal [18], and gastric cancers [19]. While certain miRNAs are upregulated [20–24] or downregulated [25–28] in CCA, these changes are not specific to this cancer type, thus necessitating the ongoing search for unique miRNA panels that improve diagnostic sensitivity and specificity.

Here, we aimed to develop a miRNA panel as a novel, effective diagnostic biomarker for CCA. We analyzed miRNA profiles using sequences of small RNAs from two genetically altered CCA cell lines (p53-mutant KKU-213B and p53-wildtype KKK-D068H1) to reduce the heterogeneity of CCA and incorporate a control of MMNK1 immortalized cholangiocyte cell line. MiRNAs upregulated in all cell lines were selected as miRNA panel. Serum samples from CCA patients and normal individuals were compared to identify a potential miRNA biomarker panel for CCA and validated with serum from HCC patients by RT-qPCR. The findings of this study demonstrate that the identified biomarker panel achieves optimal diagnostic accuracy for CCA, with excellent sensitivity, specificity, and area under the curve (AUC) values in receiver operating characteristic (ROC) analysis, highlighting its potential to improve diagnosis.

## Materials and methods

### Sample collection and ethics statement

The Japanese Collection of Research Bio-Resources (JCRB) Cell Bank in Japan provided the immortalized cholangiocyte cell line (MMNK1) and the two human CCA cell lines (KKU-213B and D068H1). The cells were cultured in Dulbecco’s modified Eagle’s medium supplemented with high glucose (4.5 g/L) (Gibco, Thermo Fisher, USA), 10% fetal bovine serum, and 1% penicillin-streptomycin. The cultures were kept at 37 °C in an incubator with 5% CO_2_. For small-RNA sequencing, frozen cell pellets were obtained from all cell lines when grown to a confluency of 50% to 70% to extract the total RNA.

Serum samples from patients diagnosed with CCA and HCC were collected at Khon Kaen University’s Cholangiocarcinoma Research Institute between 2 February 2022 and 20 December 2023. The study was approved by the Khon Kaen University Ethics Committee for Human Research (Reference No. HE 571283), and all patients provided written informed consent. This study employed leftover serum samples of those patients. Normal control (NC) samples were taken from leftover serum of healthy individuals who came to Khon Kaen University’s Faculty of Associated Medical Sciences’ Office for Medical Technology and Physical Therapy Health Service from 26 June to 22 July in the year 2023. The study protocol (HE 662104) was approved by the Khon Kaen University Human Ethics Committee. All blood was collected in clotting tubes and processed within two hours of collection. Any samples exhibiting hemolysis were discarded. After the serum was separated by centrifugation at 1500 rpm for 10 minutes at 4 °C, it was promptly aliquoted and kept at −80 ºC until miRNA extraction. Three hundred microliters of serum per sample were utilized for RNA extraction and subsequent experimental procedures.

### Small-RNA sequencing and analysis

Frozen cell pellets of three cell lines were used: p53-mutant KKU-213B CCA cells (n = 3 per cell line), p53-wildtype KKK-D068H1 CCA cells (n = 3), and MMNK1 immortalized normal cholangiocyte cells (n = 3). RNA was extracted from cell pellets using the PureLink RNA Mini Kit and subjected to small-RNA sequencing. A total of 3 μg of total RNA per sample was used as input material for the small-RNA library. Sequencing libraries were generated using the NEBNext Multiplex Small-RNA Library Prep Set for Illumina (NEB, USA.). PCR amplification was performed using LongAmp Taq 2X Master Mix, SR Primer for Illumina, and index (X) primer. Library quality was assessed on the Agilent Bioanalyzer 2100 system using DNA high-sensitivity chips. The clustering of the index-coded samples was performed on a cBot Cluster Generation System using TruSeq SR Cluster Kit v3-cBot-HS (Illumina). After cluster generation, the library preparations were sequenced on an Illumina platform, and 50-bp single-end reads were generated.

Clean data (clean reads) were obtained by removing reads containing poly-N, with 5´ adapter contaminants, without the 3´ adapter or the insert tag, containing poly A or T or G or C, and low-quality reads from raw data. The small-RNA tags were mapped to the reference sequence using Bowtie [29] without mismatches to analyze their expression and distribution. To identify known miRNAs, small-RNA tags were first filtered to remove sequences from protein-coding genes, repeats, and other non-coding RNAs (rRNA, tRNA, etc.) by mapping against the RepeatMasker and Rfam databases. The filtered tags were then aligned with the miRBase 20.0 reference using the miRDeep2 [30] and srna-tools-cli software. Custom scripts were subsequently employed to quantify miRNA read counts and analyze positional nucleotide bias. Specifically, we analyzed the base bias at the first nucleotide and across the full length of all identified miRNAs.

### Selection of candidate miRNAs

After small-RNA sequencing, seven candidate miRNAs were selected as significantly and differentially expressed according to log2 fold change (> 4.5), p-value (<0.05). Detailed information on these candidate miRNAs is presented in S1 Table.

### RNA extraction, cDNA synthesis, and quantitative real-time polymerase chain reaction

RNA was extracted from serum samples using the NucleoSpin^®^ miRNA plasma kit (Macherey-Nagel, Germany), and miRNA was reverse-transcribed to cDNA using the Agilent miRNA 1^st^-Strand cDNA Synthesis kit (USA) according to the manufacturer’s protocols. The primer sequences for the seven candidate miRNAs and a housekeeping miRNA (miRNA-16) are shown in S2 Table. RT-qPCR analysis was performed in duplicate using the miScript SYBR® Green PCR Kit and specific primers according to the manufacturer’s instructions. The RT-qPCR conditions were as follows: 95 °C for 5 min for enzyme activation, followed by 45 cycles of denaturing at 95 °C for 10 sec, primer annealing at 60 °C for 20 sec, and cDNA amplification at 72 °C for 30 sec using the LightCycler^®^ 480 Real-Time PCR System (Roche). Relative gene and miRNA expression levels were determined using RT-qPCR. Target gene and miRNA expression were normalized to the endogenous control, miRNA-16, and quantified using the 2^−ΔΔCt^ method. Each experiment was conducted twice.

### Assessing tumor markers in serum samples

Serum levels of CA19−9 and CEA were measured using an automated analyzer (Elecsys e801, part of the cobas 8000 modular analyzer series, Roche Diagnostics, Mannheim, Germany) at the clinical laboratory section of Srinagarind Hospital, Faculty of Medicine, Khon Kaen University. To ensure the precision and accuracy of the measurements, internal quality control materials (PC TM, Roche) and external quality control materials (Riqas, Randox Laboratories, Antrim, UK) were utilized.

### Functional and pathway analysis

The Kyoto Encyclopedia of Genes and Genomes pathway (KEGG) and Gene Ontology (GO) analysis were performed using Shinygo 0.82 to identify and analyze significantly upregulated miRNAs (cutoff adjusted p-value < 0.01) between p53-mutant and p53-wildtype CCA cell lines. The biological process (BP), cellular components (CC), and molecular functions (MF) of the GO terms were noted. An adjusted p-value of less than 0.05 was used to identify significant results. If more than 20 terms were found, the top 20 were shown using the techniques outlined.

### Statistical analysis

The serum miRNA expression levels of CCA patients, NC, and HCC patients were compared using the Mann-Whitney test. G*Power (version 3.1) software was used for the determination of the optimal sample size for experiments, and above 80% was obtained in each compared group. ROC curves and the area under the ROC curve were analyzed to assess the diagnostic values of the detected serum and the panel. The DeLong method was used to determine statistical differences between ROC curves [31]. Correlations between the expression levels of three potential miRNAs and patients’ clinicopathological data were assessed using Chi-square and Fisher’s exact tests. SPSS 20.0 was used to process all the data, and GraphPad Prism (version 9.0) was used to plot the results. A p-value of less than 0.05 was deemed statistically significant.

## Results

### Discovery of potential miRNAs for the diagnosis of CCA

**Fig 1:**
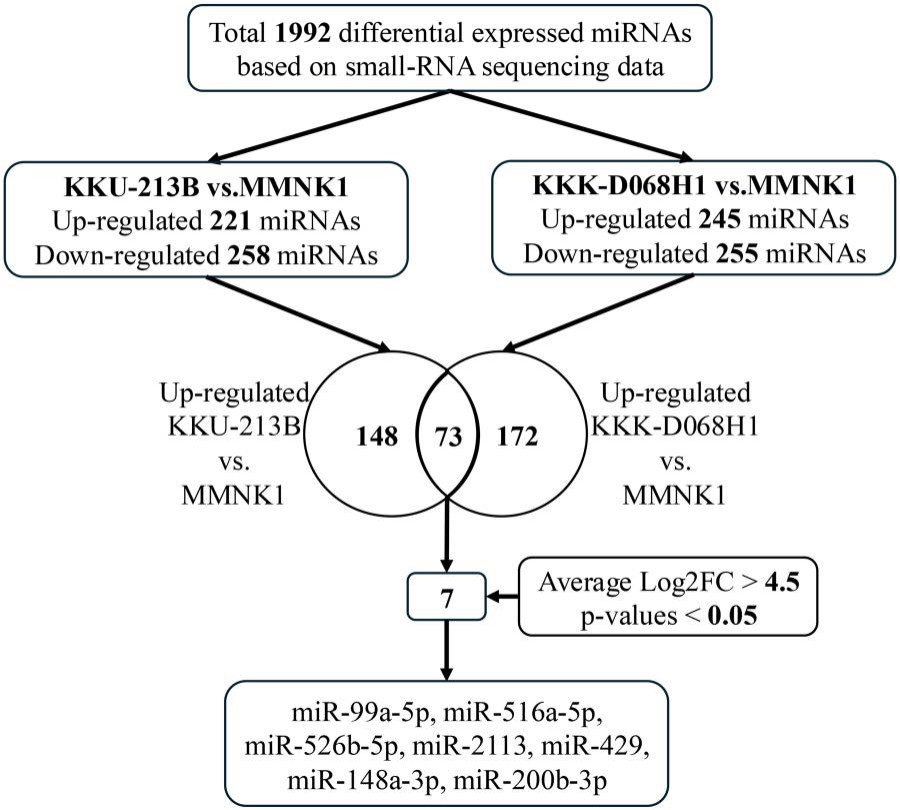
Workflow for selecting candidate miRNAs.

In this study, small-RNA sequences from three cell lines (p53-mutant KKU-213B, p53-wildtype KKK-D068H1, and MMNK1) were analyzed. In total, 1992 differentially expressed miRNAs (DE miRNAs) were detected in comparisons among the cell lines. Of these, 221 were upregulated in KKU-213B relative to MMNK1 and 245 were upregulated in KKK-D068H1 relative to MMNK1. Seventy-three miRNAs appeared in both lists. Finally, seven upregulated miRNAs with a log2 fold change (FC) > 4.5 were selected for further investigation: miR-99a-5p, miR-516a-5p, miR-526b-5p, miR-2113, miR-429, miR-148a-3p, and miR-200b-3p, as shown in Fig 1.

### Diagnostic performance for CCA of seven candidate miRNAs

**Fig 2:**
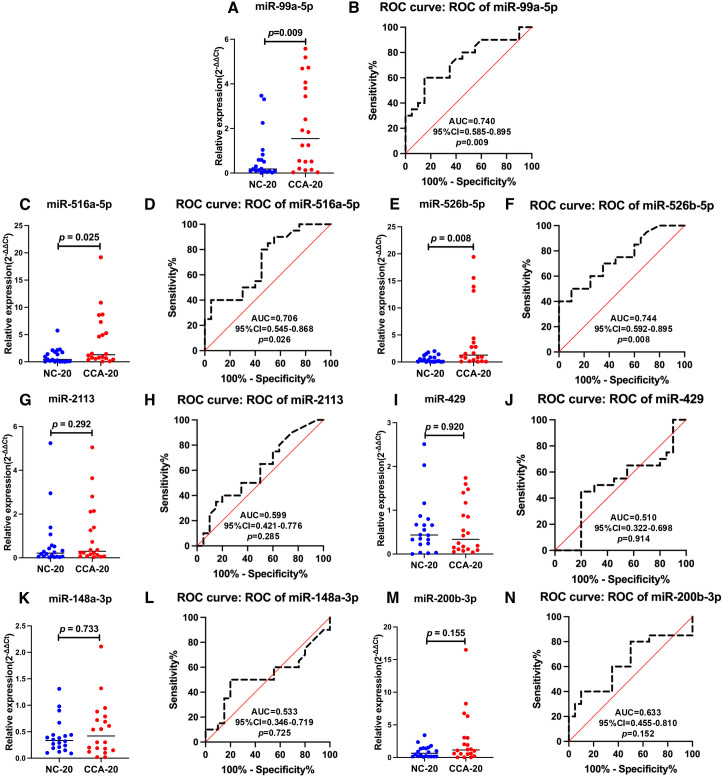
Diagnostic performance of potential miRNAs in CCA patients using relative expression and ROC curve analyses. The expression levels of seven potential miRNAs were quantified by RT-qPCR in 40 serum samples (20 NC and 20 CCA samples), and ROC curve analysis for seven miRNAs was also performed. Three candidate miRNAs—miR-99a-5p **(A, B)**, miR-516a-5p **(C, D)**, and miR-526b-5p **(E, F)**—had significant p-values <0.05 and AUC values in comparisons between healthy and CCA sera. The remaining four candidate miRNAs—miR-2113 **(G, H)**, miR-429 **(I, J)**, miR-148a-3p **(K, L)**, and miR-200b-3p **(M, N)**—did not significantly differ in expression results and had AUC values below 0.7. Sera from CCA patients revealed a considerable upregulation of three miRNAs: miR-99a-5p, miR-516a-5p, and miR-526b-5p.

To evaluate potential miRNAs to distinguish CCA patients from NC, we measured the seven candidate miRNAs in sera from both groups (n = 20 in each) using RT-qPCR. The sample sizes were adequate according to G*Power analysis (>80% statistical power). MiRNA-16 was used as an endogenous housekeeping miRNA to normalize the expression of selected miRNAs in serum [32]. We also performed an ROC-curve study to assess the probable detection threshold for the seven candidate miRNAs during screening. Three of the candidates, miR-99a-5p (Fig 2A, 2B), miR-516a-5p (Fig 2C, 2D), and miR-526b-5p (Fig 2E, 2F**),** were significantly upregulated in CCA compared to NC in serum according to RT-qPCR and ROC analysis. These three miRNAs were assessed for their ability to distinguish between patients with CCA and NC or patients with HCC. There was no discernible statistical difference between NC and CCA sera in the levels of the remaining four microRNAs: miR-2113 (Fig 2G, 2H), miR-429 (Fig 2I, 2J), miR-148a-3p (Fig 2K, 2L), and miR-200b-3p (Fig 2M, 2N).

### High diagnostic utility of three miRNAs for the diagnosis of CCA

**Fig 3:**
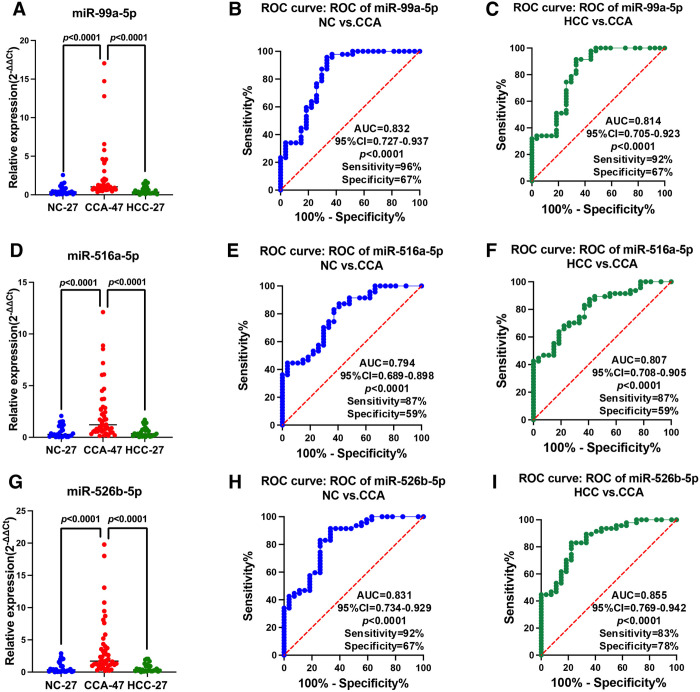
Validation of three miRNAs using relative expression and ROC curves. All three were significantly upregulated in CCA (n = 47) compared with NC (n = 27) and HCC (n = 27). The expression levels and AUC values in the CCA vs. NC and CCA vs. HCC of the three candidate miRNAs, miR-99a-5p (A, B, C), miR-516a-5p (D, E, F), and miR-526b-5p (G, H, I) were determined by RT-qPCR and ROC analysis, respectively.

The validation step utilized a greater number of CCA sera and NC sera than the screening step. Serum samples from patients with HCC were added to identify biomarkers more specific to CCA. For the validation phase, we used serum samples from 27 NC, 47 CCA patients, and 27 HCC patients. The three miRNAs we tested were those with considerably elevated expression in CCA in comparison to NC (as shown in Fig 2). When the CCA and NC groups were compared using the larger number of sera in ROC curve analysis, miR-99a-5p, miR-516a-5p, and miR-526b-5p had AUC values of 0.832, 0.794, and 0.831, respectively. In comparisons between the CCA and HCC groups, it was found that miR-99a-5p, miR-516a-5p, and miR-526b-5p had AUC values of 0.814, 0.807, and 0.855, respectively. The diagnostic utility of these miRNAs is shown in Fig 3. These findings indicate that a panel of circulating miRNAs, comprising miR-99a-5p, miR-516a-5p, and miR-526b-5p, may function as a reliable set of biomarkers for the identification of CCA.

### A circulating miRNA panel enhances the efficacy of CCA diagnosis

**Fig 4:**
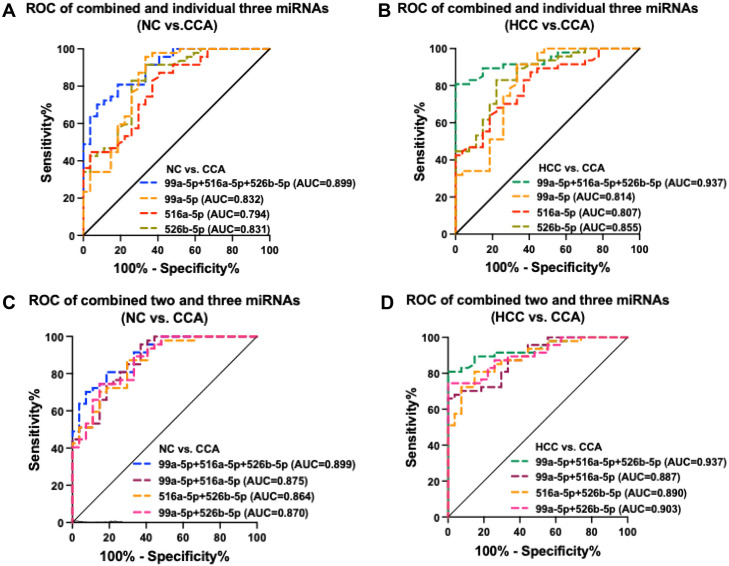
Individual candidate miRNAs alone or combined enhances the efficiency of CCA diagnosis. All three miRNAs were combined and individually for the prediction of CCA in the comparison group of **(A)** NC vs. CCA and **(B)** HCC vs. CCA. The AUC value of all three miRNAs combined was higher than the AUC values of individual miRNAs. All three combined and the three possible combinations of two miRNAs for the prediction of CCA in comparisons between **(C)** NC vs. CCA and **(D)** HCC vs. CCA.

When the three miRNAs were combined for the analysis, the sensitivity and specificity values were 81% and 82%, respectively, and the AUC was 0.899 in the CCA compared to the NC group. When the CCA group was compared with the HCC group, the sensitivity and specificity values were 81% and 100%, respectively, and the AUC was 0.937. When all three miRNAs were combined, these values were greater than for single miRNAs (Fig 4A, 4B) and the combination of two miRNAs (Fig 4C, 4D) in both analyses. Statistical comparison between the ROC curves by the DeLong method showed a significantly higher AUC value for the three-miRNA combination compared to miR-99a-5p alone (p = 0.047) and to miR-516a-5p alone (p = 0.022) in the CCA vs. HCC group (S3 Table). Table 1 displays a summary of the data (AUC values, 95% confidence intervals, cut-off values, sensitivity%, specificity%, positive predictive values, negative predictive values, accuracy%) for single miRNAs and combinations of any two and all three miRNAs.

**Table 1:** Ability of individual and combined circulating miRNAs to discriminate CCA from NC and HCC by RT-qPCR.

miRNA	AUC	95% CI	p-value	Cut off values	Sensitivity	Specificity	PPV	NPV	AC
**NC vs. CCA**									
miR-99a-5p	0.832	0.727-0.937	<0.0001	0.47	96%	67%	85%	90%	86%
miR-516a-5p	0.794	0.689-0.898	<0.0001	0.41	87%	59%	79%	73%	77%
miR-526b-5p	0.831	0.734-0.929	<0.0001	0.50	92%	67%	82%	81%	82%
miR-99a-5p + 516a-5p	0.875	0.794-0.956	<0.0001	0.39	96%	63%	80%	89%	82%
miR-516a-5p + 526b-5p	0.864	0.781-0.947	<0.0001	0.46	87%	70%	83%	73%	80%
miR-99a-5p + 526b-5p	0.870	0.788-0.952	<0.0001	0.63	75%	85%	90%	66%	78%
miR-99a-5p + 516a-5p + 526b-5p	0.899	0.831-0.967	<0.0001	0.66	81%	82%	81%	82%	78%
**HCC vs. CCA**									
miR-99a-5p	0.814	0.705-0.923	<0.0001	0.62	92%	67%	83%	82%	82%
miR-516a-5p	0.807	0.708-0.905	<0.0001	0.41	87%	59%	79%	73%	77%
miR-526b-5p	0.855	0.769-0.942	<0.0001	0.94	83%	78%	87%	72%	81%
miR-99a-5p + 516a-5p	0.887	0.815-0.958	<0.0001	0.79	68%	96%	97%	62%	77%
miR-516a-5p + 526b-5p	0.890	0.818-0.961	<0.0001	0.60	81%	85%	90%	72%	82%
miR-99a-5p + 526b-5p	0.903	0.838-0.969	<0.0001	0.72	75%	100%	97%	67%	81%
miR-99a-5p + 516a-5p + 526b-5p	0.937	0.885-0.989	<0.0001	0.72	81%	100%	81%	100%	89%

95% CI = 95% confidence interval, PPV = Positive predictive value, NPV = Negative predictive value, AC = Accuracy

### A circulating miRNA panel combined with established tumor markers improves ability to diagnose CCA patients

**Fig 5:**
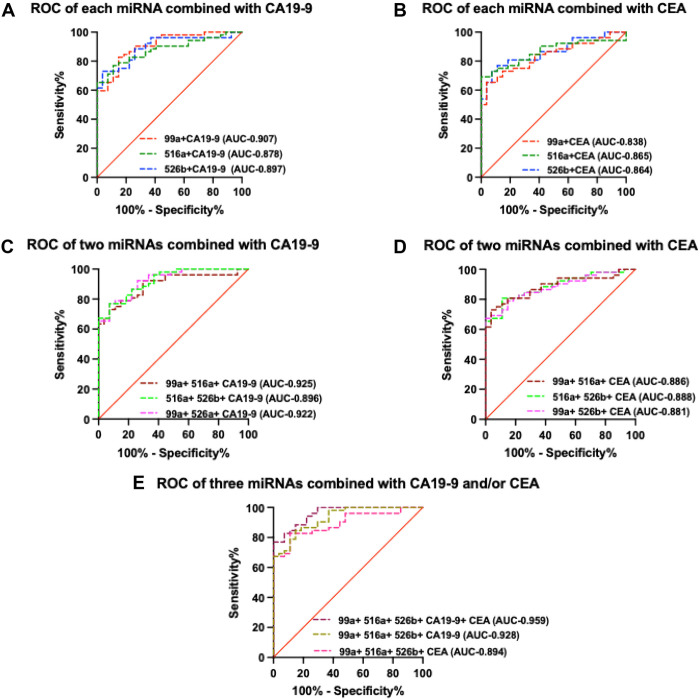
Combining miRNAs with established tumor markers improves the diagnosis of CCA. AUC values of candidate miRNAs separately combined with tumor markers CA19−9 (A) and CEA **(B)**. The same situation occurred for two of the three possible combinations of two miRNAs with CA19−9 (C) and CEA **(D)**. When all three miRNAs were combined with CA19−9 and CEA, the highest AUC value was obtained **(E)**.

Next, we checked to see if the useful established tumor markers found in CCA—CA19−9, and CEA—could improve diagnostic accuracy when combined with the three candidate miRNAs. The AUC values of the individual candidate miRNAs (miR-99a-5p, miR-516a-5p, and miR-526b-5p) combined with CA19−9 were much higher than those of individual miRNAs for the same samples (Fig 5A). A similar pattern was seen in the combination of each candidate miRNA with CEA (Fig 5B). Furthermore, two of the three possible combinations of any two miRNAs with either CA19−9 or CEA had higher AUC values relative to those of any two miRNAs (Fig 5C and 5D). The combination of all three miRNAs with CA19−9 exhibited a higher AUC value, but with CEA showed nearly the same AUC value compared with the combined three miRNAs. Finally, the combination of all three miRNAs, CA19−9, and CEA had the highest AUC value found (0.959) (Fig 5E). Evaluation of the ROC curves using the DeLong method showed that the three-miRNA combination had a significantly higher AUC value than either CA19−9 (p = 0.039) or CEA (p = 0.008). When our miRNA panel was combined with the conventional markers (CA19−9 and CEA), the performance was also significant (p = 0.035). A summary of the findings is shown in Table 2.

**Table 2:** Comparisons of individual and combined miRNAs with tumor markers in the same CCA patient cohort.

miRNA	AUC	95% CI	p*-*value	Cut off values	Sensitivity	Specificity	PPV	NPV	AC
**Candidate miRNA(s) with CA19−9**									
miR-99a-5p + CA19−9	0.907	0.844 - 0.971	<0.0001	0.48	83%	85%	91%	72%	84%
miR-516a-5p + CA19−9	0.878	0.805 - 0.952	<0.0001	0.57	77%	89%	93%	69%	82%
miR-526b-5p + CA19−9	0.897	0.829 - 0.966	<0.0001	0.72	73%	96%	97%	65%	81%
miR-99a-5p + 516a-5p + CA19−9	0.925	0.869 - 0.98	<0.0001	0.66	77%	93%	97%	65%	81%
miR-516a-5p + 526b-5p + CA19−9	0. 896	0.827 - 0.965	<0.0001	0.69	73%	93%	95%	64%	80%
miR-99a-5p + 526b-5p + CA19−9	0. 922	0.867 - 0.978	<0.0001	0.71	77%	93%	95%	68%	82%
miR-99a-5p + 516a-5p + 526b-5p + CA19−9	0.928	0.875 - 0.981	<0.0001	0.54	85%	85%	95%	64%	82%
**Candidate miRNA(s) with CEA**									
miR-99a-5p + CEA	0.838	0.753 - 0.924	<0.0001	0.74	65%	96%	97%	59%	76%
miR-516a-5p + CEA	0.865	0.785 - 0.945	<0.0001	0.77	69%	100%	100%	63%	80%
miR-526b-5p + CEA	0.864	0.786 - 0.942	<0.0001	0.66	77%	89%	93%	65%	80%
miR-99a-5p + 516a-5p + CEA	0.886	0.814 - 0.958	<0.0001	0.74	73%	96%	97%	65%	81%
miR-516a-5p + 526b-5p + CEA	0.888	0.818 - 0.958	<0.0001	0.63	81%	89%	93%	71%	84%
miR-99a-5p + 526b-5p + CEA	0.881	0.809 - 0.953	<0.0001	0.8	67%	100%	100%	61%	78%
miR-99a-5p + 516a-5p + 526b-5p + CEA	0.894	0.826 - 0.962	<0.0001	0.6	83%	89%	93%	73%	85%
**Three miRNAs with both CA19−9 and CEA**									
miR-99a-5p + 516a-5p + 526b-5p+ CA19−9 + CEA	0.959	0.922 - 0.996	<0.0001	0.86	77%	100%	100%	69%	85%

95% CI = 95% confidence interval, PPV = Positive predictive value, NPV = Negative predictive value, AC = Accuracy

### Correlation of the three potential miRNAs and clinicopathological factors in CCA patients

Chi-square and Fisher’s exact tests were used to investigate the clinical relevance of the analysis of three candidate miRNAs. The median value for 67 CCA sera was used as the cutoff to distinguish the p-value. Our study aimed to determine if age, gender, lymph-node metastasis, distant metastasis, and histological type were significantly correlated with the expression levels of three potential miRNAs. No correlations were found, as shown in S4 Table.

### Functional and pathway analyses

**Fig 6:**
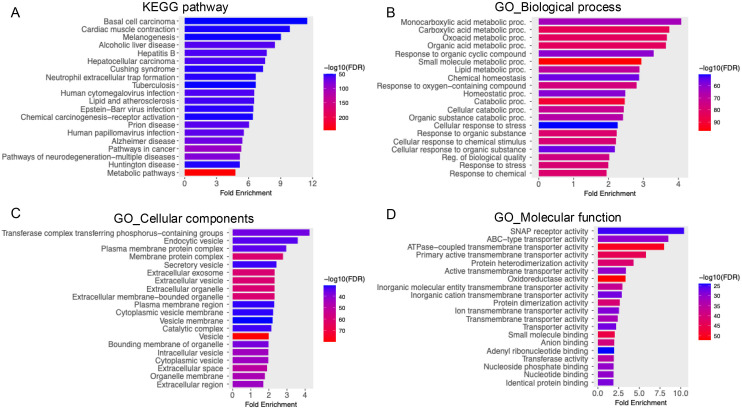
The KEGG and GO analysis of significantly upregulated miRNAs. The pathways relating to target gene enrichment in the KEGG analysis **(A)**. The biological process **(B)**, cellular components **(C)**, and molecular function (D) analyses of target gene enrichment in GO analysis.

KEGG pathway and GO analysis were performed on the predicted target genes of significantly upregulated miRNAs compared between p53-mutant and p53-wildtype cell lines. The results are shown in Fig 6. KEGG pathway analysis revealed significant enrichment in canonical cancer-related pathways, including pathways in cancer and chemical carcinogenesis-receptor activation, as well as hepatocellular carcinoma (Fig 6A). In GO enrichment, response to chemicals, response to stress, cellular response to organic substance, and cellular response to chemical stimulus were linked to the differentially expressed miRNAs using BP analysis (Fig 6B). Cellular components (CC) analysis noted changes in extracellular exosome, vesicle membrane, and plasma membrane protein complex, all suggesting a significant alteration in membrane-bound and secreted components (Fig 6C). Molecular functions (MF) analysis revealed that transmembrane transporter activities, including ABC transporters and ATPase-coupled transporters, reflect microRNA-mediated regulation of transport functions that are known to be influenced by p53 (Fig 6D). The findings strengthen the biological significance of our study’s models and indicate that dysregulated miRNAs might play a role in p53-related cellular remodeling.

## Discussion

This study reports the findings of three candidate miRNAs as potential diagnostic markers for CCA and explores how this miRNA panel can be applied to support better diagnosis of CCA, particularly in patients suspected of concurrent CCA and HCC. We discovered candidate miRNAs using small-RNA sequencing in three cell lines, namely p53-mutant, wildtype CCA, and an immortalized cholangiocyte (MMNK1) line. Seven miRNAs exhibited significant upregulation in p53-mutant and wildtype CCA cell lines relative to MMNK1. RT-qPCR was performed on the sera of CCA patients and NC, revealing significantly higher expression of three out of seven miRNAs (miR-99a-5p, miR-516a-5p, and miR-526b-5p). CCA can sometimes be challenging to differentiate from HCC because both have morphological, genomic, and clinical heterogeneity [33–35]. Hence, we further validated these three candidate miRNAs in CCA, HCC, and NC. Interestingly, the combination of two or three of these miRNAs was found to exhibit more effective diagnostic accuracy than each miRNA alone. In addition to this, integrating it with known CCA tumor markers led to much higher accuracy. Various combinations of the three candidate miRNAs and the established biomarkers CA19−9 and CEA were tested. Combining all three miRNAs with both established markers yielded the best diagnostic performance.

The selected miRNAs in our study possess scientific interest owing to their respective roles as diagnostic markers in a variety of cancers according to previous publications. One of the candidate miRNAs, miR-99a-5p, is a predictive and diagnostic biomarker in tissue samples from bladder cancer [36] and early-stage signet-ring cell carcinoma [37]. Previous studies [38,39] have demonstrated that miR-99a-5p in plasma is a novel and minimally invasive biomarker for the detection of breast cancer. It is also a biomarker in plasma extracellular vesicles for early head-and-neck squamous-cell carcinoma. Additionally, by targeting the oncogenic molecules family with sequence similarity 64 member A and cell-division cycle 25A/ interleukin 6, miR-99a-5p has been shown to operate as a tumor suppressor in human cases of cervical squamous-cell carcinoma [40] and lung adenocarcinoma [41]. Moreover, miR-99a-5p expression is downregulated in HCC, and the hsa-miR-99a-5p-TAOK1 competing endogenous RNA network has been linked to an improved prognosis for HCC patients [42]. As a tumor suppressor, miR-516a-5p targets several genes, including histone cluster 3, H2a, circ MYC proto-oncogene, BHLH transcription factor, and PBX homeobox 3 in non-small cell lung cancer [43], acute myeloid leukemia [44], and HCC [45]. The functional studies of circular RNA and long non-coding RNA in HCC [46–48], non-small cell lung cancer [49], and cervical cancer [50] suggest that miR-526b–5p may act as a “sponge” miRNA.

Our findings indicated that the expression levels of miR-99a-5p, miR-516a-5p, and miR-526b-5p were elevated only in CCA when compared to HCC and NC. A previous study reported that levels of four different miRNAs were specifically and consistently upregulated in HCC patients but not in non-HCC patients, and they concluded that these markers might be specific to HCC [51]. Comparably, a signature panel of seven distinct cell-free miRNAs was identified in a prior investigation of ovarian cancer. This panel of seven miRNAs, when combined with the current gold-standard marker CA-125, could differentiate between benign and malignant tumors and help predict early ovarian cancer [52]. Our findings demonstrate that integrating miRNA panels with established clinical markers provides a synergistic strategy for developing more effective diagnostic approaches for CCA.

Regarding the diagnosis of CCA, a circulating two-miRNA panel of upregulated miR-877 and downregulated miR-16 has been previously shown to have good potential in diagnosing individuals with distal CCA and distinguishing it from benign disease and pancreatic ductal adenocarcinoma [53]. Bile exosomal miR-200a-3p, miR-200c-3p and serum exosomal miR-200c-3p could be diagnostic biomarkers for CCA, and high levels of these markers correlate with adverse clinical outcomes [54]. Additionally, circulating miR-423-5p, miR-93-5p, and miR-4532 are promising biomarkers. For the diagnosis of CCA in Ov-endemic regions, a sequential screening approach has been recommended, which involves initial testing with miR-4532 and subsequent validation using miR-423-5p and miR-93 [55]. Another study of circulating miRNAs as diagnostic markers showed that miR-122, miR-192, miR-29b and miR-155 increased in CCA compared to NC or primary sclerosing cholangitis patients. A remarkable reduction in miR-122 level after surgery was also related to a favorable prognosis in CCA patients [56]. In the previous studies, panels of circulating miRNA play a role in the diagnosis of CCA, distinguishing CCA from normal and benign conditions, and correlating with clinical outcomes and prognosis. The novelty of this study is the detection of a new three-miRNA panel significantly increased in CCA compared to not only NC but also HCC.

While this research provides significant insights regarding miRNAs, there are a few perspectives that should be considered for further improvement and robustness. To begin with, distinct miRNA biomarkers vary based on the type of cancer and the samples’ origin, blood-cell contamination and the anticoagulant employed during collection may have an impact on plasma or serum measurements [57]. Therefore, serum samples were used in our RT-qPCR analysis to remove any potential interference from such causes. The KEGG pathway and GO enrichment analyses were performed on the predicted targets of significantly upregulated miRNAs between p53-mutant and p53-wildtype cell lines. These demonstrated the feasibility of dysregulated miRNAs as diagnostic indicators influenced by the presence of p53 mutations in both cell lines. Another thing to consider is the correlation of miRNAs and clinicopathological aspects of CCA; there was no significant correlation between three candidate miRNAs and clinicopathological parameters of CCA patients. We also investigated whether our three candidate miRNAs can distinguish CCA stages and histological types (S5 Table). However, the analysis revealed no significant differences between early and advanced stages of CCA. These findings indicated that our three miRNAs have potential as diagnostic biomarkers but may not be good prognostic indicators. Similar to our study, upregulation of miR-18a and miR-532 were used as a potential biomarker in extrahepatic CCA but showed no correlation with tumor markers or histopathological grading [58]. The role of miRNAs as biomarkers is often highly histotype specific. For instance, miR-205 has been identified as a specific marker for differentiating squamous from non-squamous subtypes of non-small cell lung cancer [59]. Similarly, in ovarian cancer, the expression levels of only some miRNAs have been directly associated with specific clinicopathological features of the disease [60].

Based on our study, a novel panel of three circulating miRNAs can be used as a diagnostic marker and it can distinguish CCA from the NC and HCC groups. To improve the diagnosis of CCA, we exclusively compared the NC group with the CCA group using our panel of miRNAs and established tumor markers. A limitation of this study is that our sample size was relatively small during the screening and validation phases. Hence, further research with a larger, multi-center prospective study is needed to explore the value of unique panels of markers for the study of certain types of cancers. In our study, additional reference miRNAs could not be used in validation because of limitations in sample availability and resources. Future investigations should utilize additional reference miRNAs to enhance the normalization accuracy and ensure the robustness of RT-qPCR results.

## Conclusion

We conclude that a panel of three specific circulating miRNAs (miR-99a-5p, miR-516a-5p, and miR-526b-5p) might be useful as minimally invasive, novel indicators for accurate diagnosis to differentiate persons with CCA not only from healthy individuals but also from HCC cases. Additionally, the diagnostic accuracy, sensitivity, and specificity of the miRNA panel and the combination of this panel with CA19−9 or CEA are higher than those of the tumor markers alone or of individual miRNAs. Further research could focus on evaluating the clinical utility of these miRNAs in larger cohorts, including confirming their specificity against other relevant diseases and pathological conditions, and investigating their roles in the pathophysiological mechanisms of CCA progression.

## Supporting information

S1 Table Small-RNA sequencing information of the selected seven candidate miRNAs. (DOCX)

S2 Table Primer sequences of candidate miRNAs and a housekeeping gene. (DOCX)

S3 Table Comparison of the ROC between candidate miRNAs and tumor markers. (DOCX)

S4 Table Correlation between the clinicopathological characteristics of individuals with CCA and the levels of expression of three potential miRNAs. (DOCX)

S5 Table The staging and histological types of CCA and HCC patients. (DOCX)
