# Supplementary material for: A circulating microRNA panel enhances the diagnosis of cholangiocarcinoma
Source: PLoS One. 2025 Sep 25;20(9):e0333279. doi: 10.1371/journal.pone.0333279 (PMC12463250; doi:10.1371/journal.pone.0333279)
Supplement: S1 Table — (DOCX) [file pone.0333279.s001.docx]

**S1 Table.** **Small-RNA sequencing information of the selected seven candidate miRNAs**

| **No** | **sRNA** | **M214 & DO68H1 readcount** | **MMNK readcount** | **log2FC** | **pval** | **padj** | **Average Log2FC** |
| --- | --- | --- | --- | --- | --- | --- | --- |
| 1 | hsa-miR-99a-5p | 32668.3025 | 1906.710911 | 4.0172 | 1.60E-39 | 2.79E-38 | 4.9876 |
|  |  | 146323.81 | 2258.745781 | 5.958 | 3.37E-152 | 5.62E-150 |  |
| 2 | hsa-miR-516a-5p | 3891.52409 | 0.509244087 | 10.946 | 1.56E-62 | 5.11E-61 | 7.21115 |
|  |  | 16.6795352 | 0.593052522 | 3.4763 | 0.00031829 | 0.0011654 |  |
| 3 | hsa-miR-526b-5p | 17176.0762 | 4.085431868 | 10.975 | 1.31E-78 | 7.57E-77 | 6.49925 |
|  |  | 24.8366193 | 4.755821746 | 2.0235 | 0.010411 | 0.026493 |  |
| 4 | hsa-miR-2113 | 421.1732 | 0.260361629 | 8.0347 | 1.01E-23 | 1.25E-22 | 7.9036 |
|  |  | 251.837844 | 0.302227047 | 7.7725 | 9.77E-25 | 1.63E-23 |  |
| 5 | hsa-miR-429 | 1579.06324 | 1.331151336 | 9.4035 | 4.32E-70 | 2.15E-68 | 7.33035 |
|  |  | 89.4923267 | 1.565869691 | 5.2572 | 1.81E-16 | 2.03E-15 |  |
| 6 | hsa-miR-148a-3p | 225893.965 | 4090.635495 | 5.5029 | 6.54E-29 | 1.34E-27 | 5.53575 |
|  |  | 206394.513 | 3424.878162 | 5.5686 | 1.17E-27 | 1.59E-26 |  |
| 7 | hsa-miR-200b-3p | 137237.191 | 679.6631924 | 7.5382 | 3.47E-170 | 2.16E-167 | 5.3923 |
|  |  | 7776.55894 | 804.7521741 | 3.2464 | 1.42E-55 | 5.51E-54 |  |

(Log2FC = log2 fold change, pval = p-value, padj = adjusted p-value)
