# Supplementary material for: A circulating microRNA panel enhances the diagnosis of cholangiocarcinoma
Source: PLoS One. 2025 Sep 25;20(9):e0333279. doi: 10.1371/journal.pone.0333279 (PMC12463250; doi:10.1371/journal.pone.0333279)
Supplement: S2 Table — (DOCX) [file pone.0333279.s002.docx]

**S2 Table. Primer sequences of candidate miRNAs and a housekeeping gene**

| miRNAs | Mature miRNAs sequences (5´ - 3´) | Primer sequence of miRNAs (5´ - 3´) |
| --- | --- | --- |
| Candidate miRNAs | | |
| miR-2113 | AUUUGUGCUUGGCUCUGUCAC | ATTTGTGCTTGGCTCTGTCAC |
| miR-429 | UAAUACUGUCUGGUAAAACCGU | TAATACTGTCTGGTAAAACCG T |
| miR-516a-5p | UUCUCGAGGAAAGAAGCACUUUC | TTCTCGAGGAAAGAAGCACTTTC |
| miR-526b-5p | CUCUUGAGGGAAGCACUUUCUGU | CTCTTGAGGGAAGCACTTTCTGT |
| miR-148a-3p | UCAGUGCACUACAGAACUUUGU | TCAGTGCACTACAGAACTTTGT |
| miR-200b-3p | UAAUACUGCCUGGUAAUGAUGA | TAATACTGCCTGGTAATGATGA |
| miR-99a-5p | AACCCGUAGAUCCGAUCUUGUG | AACCCGTAGATCCGATCTTGTG |
| Housekeeping miRNA | | |
| miR-16 | UAGCAGCACGUAAAUAUUGGCG | TAGCAGCACGTAAATATTGGCG |
