# Supplementary material for: A circulating microRNA panel enhances the diagnosis of cholangiocarcinoma
Source: PLoS One. 2025 Sep 25;20(9):e0333279. doi: 10.1371/journal.pone.0333279 (PMC12463250; doi:10.1371/journal.pone.0333279)
Supplement: S3 Table — (DOCX) [file pone.0333279.s003.docx]

**S3 Table. Comparison of the ROC between candidate miRNAs and tumor markers**

| **Marker 1** | **Marker 2** | **SE different** | **Z score** | **p-value** |
| --- | --- | --- | --- | --- |
| **CCA vs. NC** | | | | |
| miR 99a-5p+516a-5p+526b-5p | miR 99a-5p | 0.0644 | 1.0468 | 0.2952 |
| miR 99a-5p+516a-5p+526b-5p | miR 516a-5p | 0.0639 | 1.6536 | 0.0982 |
| miR 99a-5p+516a-5p+526b-5p | miR 526b-5p | 0.0609 | 1.1129 | 0.2658 |
| miR 99a-5p+516a-5p+526b-5p | miR 99a-5p+516a-5p | 0.0541 | 0.4511 | 0.6519 |
| miR 99a-5p+516a-5p+526b-5p | miR 99a-5p+526b-5p | 0.0545 | 0.535 | 0.5926 |
| miR 99a-5p+516a-5p+526b-5p | miR 516a-5p+526b-5p | 0.055 | 0.6452 | 0.5188 |
| miR 99a-5p+516a-5p+526b-5p | CA19-9 | 0.0641 | 2.0623 | **0.0392** |
| miR 99a-5p+516a-5p+526b-5p | CEA | 0.066 | 2.6375 | **0.0084** |
| miR 99a-5p+516a-5p+526b-5p | CA19-9+CEA | 0.0514 | 0.2769 | 0.7818 |
| miR 99a-5p+516a-5p+526b-5p+  CA19-9+CEA | miR 99a-5p+  516a-5p+526b-5p | 0.0379 | 1.8992 | 0.0575 |
| miR 99a-5p+516a-5p+526b-5p+  CA19-9+CEA | CA19-9+CEA | 0.0409 | 2.1078 | **0.035** |
| **CCA vs. HCC** | | | | |
| miR 99a-5p+516a-5p+526b-5p | miR 99a-5p | 0.062 | 1.9812 | **0.0476** |
| miR 99a-5p+516a-5p+526b-5p | miR 516a-5p | 0.0569 | 2.2851 | **0.0223** |
| miR 99a-5p+516a-5p+526b-5p | miR 526b-5p | 0.0518 | 1.5667 | 0.1172 |
| miR 99a-5p+516a-5p+526b-5p | miR 99a-5p+516a-5p | 0.0451 | 1.1085 | 0.2676 |
| miR 99a-5p+516a-5p+526b-5p | miR 99a-5p+526b-5p | 0.0426 | 0.7863 | 0.4317 |
| miR 99a-5p+516a-5p+526b-5p | miR 516a-5p+526b-5p | 0.0452 | 1.0374 | 0.2996 |

The comparison of the AUC in ROC between candidate miRNAs and tumor markers was done using the DeLong method in EasyROC (version 1.3.1). (SE = standard error)
