# Supplementary material for: A circulating microRNA panel enhances the diagnosis of cholangiocarcinoma
Source: PLoS One. 2025 Sep 25;20(9):e0333279. doi: 10.1371/journal.pone.0333279 (PMC12463250; doi:10.1371/journal.pone.0333279)
Supplement: S4 Table — (DOCX) [file pone.0333279.s004.docx]

**S4 Table. Correlation between the clinicopathological characteristics of individuals with CCA and the levels of expression of three potential miRNAs.**

| **Variable** |  | **n** | **miR-99a-5p** | | p-value | **miR-516a-5p** | | p-value | **miR-526b-5p** | | p-value |
| --- | --- | --- | --- | --- | --- | --- | --- | --- | --- | --- | --- |
|  |  |  | Low(n) | High(n) |  | Low(n) | High(n) |  | Low(n) | High(n) |  |
| **Age (years)** | < 67 | 33 | 19 | 14 | 0.914 | 19 | 14 | 0.179 | 15 | 18 | 0.540 |
|  | ≥ 67 | 34 | 14 | 20 |  | 14 | 20 |  | 18 | 16 |  |
| **Gender** | Male | 43 | 20 | 23 | 0.179 | 20 | 23 | 0.548 | 22 | 21 | 0.676 |
|  | Female | 24 | 13 | 11 |  | 13 | 11 |  | 11 | 13 |  |
| **Lymph node metastasis** | |  |  |  |  |  |  |  |  |  |  |
| Yes | | 33 | 16 | 17 | 0.901 | 15 | 18 | 0.540 | 15 | 18 | 0.540 |
| No | | 34 | 17 | 17 |  | 18 | 16 |  | 18 | 16 |  |
| **Distance metastasis** | |  |  |  |  |  |  |  |  |  |  |
|  | Yes | 14 | 4 | 10 | 0.082 | 8 | 6 | 0.507 | 8 | 6 | 0.507 |
|  | No | 53 | 29 | 24 |  | 25 | 28 |  | 25 | 28 |  |
| **Histological type** | |  |  |  |  |  |  |  |  |  |  |
|  | Non-papillary | 44 | 24 | 20 | 0.231 | 21 | 23 | 0.730 | 23 | 21 | 0.494 |
|  | Papillary | 23 | 9 | 14 |  | 12 | 11 |  | 10 | 13 |  |

The clinicopathological data and expression of three potential miRNAs in sera were analyzed using Chi-square and Fisher's exact tests. p*-*value was determined with the Pearson Chi-square test. **p*<0.05.
