# Supplementary material for: A circulating microRNA panel enhances the diagnosis of cholangiocarcinoma
Source: PLoS One. 2025 Sep 25;20(9):e0333279. doi: 10.1371/journal.pone.0333279 (PMC12463250; doi:10.1371/journal.pone.0333279)
Supplement: S5 Table — (DOCX) [file pone.0333279.s005.docx]

**S5 Table.** **The staging and histological types of CCA and HCC patients.**

| Stages | CCA (n = 67) | | | HCC (n = 27) |
| --- | --- | --- | --- | --- |
|  | Intra-hepatic | Perihilar | Extra-hepatic |  |
| I | 7 | - | - | 5 |
| II | 9 | - | 2 | 14 |
| III | 8 | 2 | 3 | 7 |
| IV | 32 | 3 | 1 | 1 |
| Total | 56 | 5 | 6 | 27 |
